# Empathy in Preschoolers: Exploring Profiles and Age- and Gender-Related Differences

**DOI:** 10.3390/children10121869

**Published:** 2023-11-29

**Authors:** Poline Simon, Nathalie Nader-Grosbois

**Affiliations:** Psychological Sciences Research Institute, UCLouvain, 1348 Ottignies-Louvain-la-Neuve, Belgium; poline.simon@uclouvain.be

**Keywords:** empathy, preschool age, profile, longitudinal

## Abstract

Empathy is a key skill in the daily life of preschoolers, and it is important to understand how it evolves during this crucial period of development. This paper includes two studies. The first study, which had a cross-sectional design, examined affective, cognitive, and behavioral empathy in 354 children (aged from 3 to 6 years) through a performance-based measure and questionnaires completed by their mothers. Although girls tended to have better affective empathy than boys on the performance-based task, no difference was noted in the mothers’ perceptions of their children’s empathy. Empathy dimensions varied depending on the age of the children. The hierarchical cluster analyses of the cases identified differentiated subgroups of children, according to their empathic skills in the three dimensions. The second study, which was longitudinal, used the same measures and investigated the developmental trajectory of empathy in 69 preschoolers over one year. The results showed that each empathic dimension predicted itself one year later, but did not predict the two others. The implications for potential interventions are emphasized in this study.

## 1. Introduction

At preschool age, it is common to see children who seem to be concerned when they witness the emotions of another child or adult. They may show sadness in response to a child who is crying, or happiness for another child who has done a beautiful drawing for his or her mother. Moreover, preschoolers can display prosocial behaviors to help or comfort a child in distress. These reactions relate to empathy, defined as an emotional response which comes from the sharing and the understanding of an emotional situation experienced by another person [1]. This emotional response is similar to that felt by the person experiencing the situation, in contrast with sympathy, which is defined as concern or sorrow felt for the condition of another person [1]. Long considered as a one-dimensional construct, empathy is now viewed as a multidimensional concept. In the literature, affective and cognitive empathy are the most commonly considered dimensions. Affective empathy is defined as the capacity to share and feel the emotion felt by another person [2], and consists of an automatic response based on an emotional stimulus, such as, for example, a facial expression, a sound, or a situation [3]. Cognitive empathy, considered as a controlled response [3], concerns the understanding of the emotional situation of others [2]. In other words, cognitive empathy corresponds to the ability to take another person’s perspective and to recognize the emotions and the social cues corresponding to an emotional situation [4]. More recently, an increasing number of authors have integrated behavioral empathy as another dimension of this concept. This refers to the different socially adapted behaviors (e.g., comforting, sharing, reassuring) triggered by the emotional situation [5]; children with behavioral problems (such as aggressiveness or opposition) find it difficult to display these behaviors.

Researchers have investigated the development of empathy as it relates to a child’s characteristics, notably gender and age. With regard to gender, the findings of some studies have revealed that girls and boys show similar levels of empathic skills, both when empathy is assessed globally [6] and when the dimensions of empathy are distinguished [7,8]. Conversely, other studies have identified a difference in global empathy [9,10], or in its affective and cognitive dimensions, in favor of girls [11]. As children usually learn their social roles by referring to adults’ expectations, which are sometimes differentiated according to gender, this may influence the development of empathy. Girls may therefore be more concerned about the emotions of others and act more prosocially when compared to boys [12]. More recent studies have nuanced their conclusions, depending on the empathic dimension considered. For example, Howe, Pit-ten Cate [13] stated that girls exhibit better cognitive empathy than boys, but that their affective empathy is equivalent. Conversely, Simon and Nader-Grosbois [14] demonstrated that cognitive empathy is higher in boys, while affective and behavioral empathy are similar between genders. Finally, Bensalah, Caillies, and Anduze [15] identified a difference in behavioral empathy only in favor of girls, which is partly in line with the conclusion of Strauss [12].

The question about the evolution of empathy with age has also been investigated at the preschool age, but the findings have been contradictory, specifically for affective empathy. According to Hoffman [16], affective empathy develops from the early months of life. However, some authors have concluded that this empathic dimension remains stable at preschool and school age [13,15,17,18], while others have found that affective empathy continues to evolve with age [7,19,20]. Cognitive empathy principally develops during the preschool period [21,22], but continues to develop throughout childhood and adolescence [13,23]. Studies regarding the relationship between age and cognitive and behavioral empathy have shown that these two skills increase as children get older, especially from preschool age on [13,15,19,20,24]. However, Nader-Grosbois and Simon [20] only observed a difference in cognitive empathy between the youngest children (3–5 years old) and the oldest children (9–12 years old). No difference was reported between the group of children aged 3–5 years and those aged 6–8 years, indicating that cognitive empathy remains stable at preschool age and during the early school years. Although a link between age and empathic skills has been investigated at preschool age, all these studies were correlational. To our knowledge, no study has ever used a longitudinal design to assess the developmental trajectory of empathy over time at preschool age. Although Nader-Grosbois and Simon [20] showed, in a transversal study, that the three dimensions of empathy were related to each other, no study has examined how accurately each dimension can be used to predict the level of the other dimensions, or its own level, after one year. Moreover, no research has ever considered whether specific skill profiles appear in the three dimensions of empathy between the ages of 3 and 6 years. It can be expected that some children with a high level of affective empathy will also have high levels of skills in regards to cognitive and behavioral empathy. Conversely, difficulties in one of the dimensions may also be related to difficulties in another. Throwing light on empathic difficulties at preschool age could be useful to prevent children from experiencing further social and emotional difficulties.

The divergent results of earlier studies may be explained by the different methods used. First, the measures used in these studies were of different types. Some studies only used hetero-reported questionnaires completed by one of the parents [9,20], while others administered a performance-based task to their participants, e.g., [13,15]. The use of multiple methods of assessment to understand children’s empathic skills is advantageous. A questionnaire completed by familiar adults reveals their perceptions of preschoolers’ empathy, based on observation in real-life situations and various social environments of children’s daily life, and thus corresponds to “implicit applied empathy”. Performance-based measures, on the other hand, can be used to directly collect data about the children’s abilities to respond to specific tasks, inspired by social situations, in regards to what they feel and think, as well as their desire to help when they are confronted by emotions expressed by another child or an adult in a fictional context. These fictional contexts are less varied and simpler than are real social situations. This type of measure reflects “explicit empathy” displayed in tasks. Second, the age ranges of the samples of children varied from one study to another. Some of them comprised only the preschool period, such as those of by Bensalah, Caillies, and Anduze [15] or Simon and Nader-Grosbois [14], while others included a larger range, from preschool to school age (e.g., 3 to 12 years old, in Nader-Grosbois and Simon [19]). Such differences in age could lead authors to differing conclusions. In addition to these two methods of evaluation, some authors have conducted studies using physiological measures, such as pupillometry, heart rate, or skin conductance, e.g., [25,26,27], to assess empathy. Although this type of measure has several advantages for assessing empathy independently of cognitive and language skills and social desirability bias [26], some limitations must be considered in its use at preschool age. For example, physiological instruments could involve difficulties linked to the availability and ease of use of the equipment [28], and their use could be frightening to children [29]. Moreover, a non-emotional stimulus could be interfered with during the evaluation [29]. It could also be difficult to distinguish whether reactions to others’ emotions reveal empathy or another process [28]. It therefore seemed difficult to implement such a method in the context of children’s everyday-life, even though physiological measures seem to be a robust method for assessing empathy [30]. Given these methodological issues, it seems relevant to combine methods which assess both implicit and explicit empathy at a specific period of preschool age.

Although several studies have investigated the question of gender or age differences in regards to empathy at preschool age, these did not apply a multi-method assessment of the three dimensions. Most of these studies (except [9]) included small (39 in [13]) or medium sample sizes (158 children in [15]). These methodological differences have prevented any consensus from being reached in regards to gender and age variability. Most studies conducted using children have led to general conclusions, without focusing on specific individual characteristics. To our knowledge, no study has explored the possibility of detecting a subgroup at risk of difficulties in regards to empathy by using questionnaires and performance-based measures. Therefore, extracting specific subgroups in a sample of typically developing preschoolers could highlight the value of taking preventive action for children with empathic difficulties. We considered it important to investigate these questions using two types of measures to assess explicit empathy (through a performance-based measure) or applied empathy in a daily-life context (with hetero-reported questionnaires) in a relatively large sample of children aged from 3 to 6 years. Exploring whether the cluster hierarchical analysis of cases could identify several subgroups of children, including those at risk, depending on their scores in each dimension of empathy, assessed by parental questionnaires and a performance-based measure, was also considered relevant. Identifying children with empathic difficulties at an early age could make it possible to implement intervention to prevent the development of social problems. The first cross-sectional study targeted these questions. In addition, no longitudinal study has previously been conducted to trace the developmental trajectory of empathy at preschool age. Although some studies have demonstrated that the three components of empathy are linked with each other, they have not shown whether these relationships persist over time. The second longitudinal study aimed to apprehend the predictive links between early empathic skills (Time 1) and those observed one year later (Time 2). Moreover, the inter-relational dynamic between affective, cognitive, and behavioral empathy at both time periods were investigated.

## 2. Study 1

### Objectives

The aim of this cross-sectional study was to investigate children’s empathy profiles at preschool age using two methods of assessment. First, this study sought to examine how the affective, cognitive, and behavioral empathic skills of preschoolers differ between boys and girls. In view of the previous literature, it was hypothesized that, across the three dimensions, children’s empathic skills would be found to vary according to their gender. Second, this study explored how affective, cognitive, and behavioral empathy evolves in children aged between 3 and 6 years. It was expected that affective empathy would remain stable over time, while cognitive and behavioral empathy would improve with age. Third, the study sought to examine whether distinct profiles of empathy could be identified, depending on children’s skills in regards to the three dimensions of empathy. It was expected that differentiated subgroups could be identified, with children with a high level of empathy in the three dimensions differing from other groups of children with lower empathic skills in one or another dimension. A further objective in this context was to determine whether any at risk subgroups could be detected among these subgroups.

## 3. Method

### 3.1. Participants

As inclusion criteria, French-speaking children aged between 3 and 6 years and without any developmental disorders were included in this study. Preschoolers who showed developmental delays or disorders (e.g., intellectual disabilities, autism spectrum disorders, language delay, or behavior problems) were excluded from the study. On the basis of these criteria, 354 preschoolers (201 girls and 153 boys) were recruited from kindergartens in the French-speaking area of Belgium. Their mean age was 4.40 years (SD = 1.028 years). To characterize the sample of this study, several items of demographic data were collected. In terms of family composition, 16.5% of the participants were only children, 55% had one sibling, and the remaining 28.5% had at least two brothers or sisters; 87% lived with both parents, while 13% lived alternately or principally with their mother or their father. Concerning socioeconomic status, Table 1 shows percentage figures indicating parents’ education level and family income. The average education level of the mothers corresponded to a bachelor’s degree, while that of fathers was equivalent to high school. The sample’s mean family income fell within the range of EUR 3500–3999 per month.

### 3.2. Measures

#### 3.2.1. Griffith Empathy Measure

The French version of the Griffith Empathy Measure GEM, [4,20] was used to assess the children’s affective and cognitive empathy, as perceived by their mothers. Using 17 items, including 13 items for affective empathy and 4 items for cognitive empathy, mothers indicated their level of agreement regarding their children’s empathic reactions in daily life situations by employing a 9-point Likert scale, from “strongly disagree” (−4) to “strongly agree” (4). In regards to affective empathy, the items of the GEM subscale related to the parental perceptions of the children’s emotion based on visible reactions, such as facial expression or vocalizations (e.g., “My child seems to react to the moods of people around him/her”). Questions regarding cognitive empathy focused on the parents’ perceptions of their children’s understanding of other people’s emotions (e.g., “My child rarely understands why other people cry”). For the two subscales, a mean score out of 4 points could be calculated. The French validation of the GEM revealed a strong internal consistency according to the values of Cronbach’s alphas for affective empathy (α = 0.82), but a less robust consistency for cognitive empathy (α = 0.61). In the present study, internal consistency was found to be acceptable for affective empathy (α = 0.76), but was still limited for cognitive empathy (α = 0.55). This meant that the results regarding cognitive empathy needed to be interpreted with caution.

#### 3.2.2. Prosocial Actions Subscale of the Empathy Questionnaire

The French version of the Empathy Questionnaire (EmQue, Rieffe, Ketelaar [19,31]) is used to evaluate the mothers’ perceptions of their children’s emotional contagion, attention to others’ feelings, and prosocial actions. In this study, only the prosocial actions subscale was used to assess behavioral empathy. On a 4-point Likert scale, ranging from “never” (0) to “always” (4), mothers indicated the frequency of their children’s day-to-day reactions and behaviors in response to the emotions of another person. A mean score out of 4 points was calculated. The Frenchvalidation of this questionnaire showed strong internal consistency according to Cronbach’s alpha values (α = 0.81). The internal consistency of the prosocial actions subscale in the present study was also strong (α = 0.80).

#### 3.2.3. Empathy Task

The Empathy Task test, created by Bensalah, Caillies, and Anduze [15], was administered to the children to evaluate their affective, cognitive, and behavioral empathy through eight stories eliciting sadness, fear, happiness, or anger (two stories for each emotion). After the different stories had been narrated, the children were asked a series of questions, each worth one point. Firstly, in a control question, the children were asked to retell the story to check their understanding. Secondly, affective empathy was assessed by asking the children about the emotions they felt when listening to the story. If the children’s emotion mirrored that of the character in the story, they gained a point. Thirdly, to assess cognitive empathy, the children were asked to explain why they felt that emotion. Finally, behavioral empathy was assessed by asking the children what actions they would take if they were in the presence of the story character. On each empathy subscale, children could accumulate a maximum of eight points. It is important to note, however, that points for cognitive and behavioral empathy were only awarded if children answered the affective empathy question. The reliability of the assessments was found to be acceptable on the basis of Cronbach’s alpha values for affective empathy (α = 0.75) and for behavioral empathy (α = 0.78). The internal consistency was strong for cognitive empathy (α = 0.84).

### 3.3. Procedure

This research project was validated by the ethical committee of the Hospital-Faculty Ethics Committee of Saint-Luc-UCLouvain. Managers of kindergartens in the French speaking area of Belgium were then approached with the purpose of recruiting participants. An information letter stating the objective of the study and a consent form were distributed to the parents. After parental consent had been received, the administration of the Empathy Task test was arranged, either at school or at home. In parallel, mothers completed the GEM-vf and the prosocial actions subscale of the EmQue-vf. Finally, the children and mothers received a gift to thank them for their participation.

## 4. Data Analysis

First, a missing data analysis and imputation were performed to improve the study’s statistical power. Second, to explore significant gender differences in regards to the three dimensions of empathy, two one-way MANOVAs were performed, using IBM SPSS 27. The first one-way MANOVA concerned the difference in the mothers’ perceptions of their children’s empathy, while the second included the three subscales of the Empathy Task. Then, the relationship between age and empathy was investigated through correlational analysis, using Pearson’s correlation coefficient. To complete this analysis, a one-way MANOVA was performed to analyze the differences between each year of age. These analyses compared empathic skills at each year of age with these skills at the other years of age. Third, two hierarchical cluster analyses of the cases were applied to extract specific subgroups of children, depending on their empathic skills in the three dimensions, using Ward’s method and squared Euclidean distance. In the same manner as the one-way MANOVAs, the first hierarchical cluster analysis of cases concerned the empathy questionnaire completed by the mothers, while the second comprised the performance-based measure of empathy. Two one-way MANOVAs were then performed to assess the difference between each subgroup of children in order to characterize their empathic skills.

## 5. Results

### 5.1. Preliminary Analysis

To improve the statistical power, a missing data analysis was performed, screening the different variables. The mean percentage of missing data through the use of variables was 17.7% (range = 11.0–29.9%). In addition, the pattern of missingness was completely random (Little’s MCAR test: χ^2^ =10.361, DF = 20, *p* = 0.961). The missing values were imputed, using expectation maximization imputation. Based on skewness and kurtosis values, the data showed a normal distribution. Table 2 presents the descriptive statistics.

Table 3 shows the zero-order correlations between the empathy measures. The Pearson’s correlation coefficients between the three dimensions of the questionnaires and those of the performance-based measure were all zero (*r* between −0.059 and 0.095; *p* between 0.060 and 0.829). Regarding the correlations between the dimensions of the mothers’ questionnaires, affective empathy was positively correlated with cognitive empathy (*r* = 0.172; *p* = 0.001) and behavioral empathy (*r* = 0.330; *p* < 0.001). Cognitive empathy was also positively related to behavioral empathy (*r* = 0.164; *p* = 0.002). Regarding the task, the three subscales positively correlated with each other (*r* between 0.600 and 0.744; *p* < 0.001).

### 5.2. Empathy Differences Depending on Gender and Age

Regarding gender differences, the first one-way MANOVA, including the three dimensions of empathy as perceived by children’s mothers, did not show a significant effect of gender (Pillai’s *F* = 0.688, *p* = 0.562, *η_p_*^2^ = 0.006). Moreover, tests of between-subjects effects did not demonstrate a significant effect of children’s gender on affective, cognitive, or behavioral empathy (*F* between 0.119 and 1.018, *p* between 0.314 and 731). The second one-way MANOVA comprised the three subscales of the performance-based measure and did not show a significant effect of gender (Pillai’s *F* = 1.310, *p* = 0.271, *η_p_*^2^ = 0.011). However, tests of between-subjects effects showed a marginal effect for affective empathy (*F =* 3.758, *p =* 0.053, *η_p_*^2^ = 0.010), in favor of girls (see Table 2 for mean values).

Regarding the relationship between age and empathy, the correlation coefficients were all significant for the variables of the questionnaires and the performance-based measure (*r* between 0.162 and 0.342, *p* between 0.000 and 0.002), except for the relationship between age and cognitive empathy, as perceived by mothers (*r = 0*.067, *p* = 0.202). Two one-way MANOVAs were then performed to identify empathy differences, according to the children’s ages. The first, including affective, cognitive, and behavioral empathy as perceived by the mothers, showed a significant effect of age (Pillai’s *F* = 2.554, *p* = 0.007, *η_p_*^2^ = 0.021). In line with the correlation coefficients, tests of between-subject effects demonstrated an effect of age for affective and behavioral empathy. More precisely, Bonferroni’s post-hoc analysis revealed a difference between children aged 3 and 6 years (mean difference = −0.48, *p* = 0.007) for affective empathy. For behavioral empathy, 3-year-old children differed from 4-year-old children (mean difference = −0.19, *p* = 0.036) and from 6-year-old children (mean difference = −0.32, *p* < 0.001). The second one-way MANOVA, including subscales of the empathy task, revealed a significant effect of age (Pillai’s *F* = 6.367, *p* < 0.001, *η_p_*^2^ = 0.050). Moreover, the tests of between-subjects effects also showed differences between the three subscales (*F* = 6.282, *p* < 0.001, *η_p_*^2^ = 0.050 for affective empathy, *F* = 17.382, *p* < 0.001, *η_p_*^2^ = 0.127 for cognitive empathy, *F* = 13.698, *p* < 0.001, *η_p_*^2^ = 0.102 for behavioral empathy). For affective empathy, Bonferroni’s post-hoc test revealed a difference between 3-year-old children and 5-year-old children (mean difference = −1.02, *p* = 0.004) and between 3-year-old children and 6-year-old children (mean difference = −1.33, *p* < 0.001). Regarding cognitive empathy, all age groups differed between each other (mean difference between −0.86 and −2.43, *p* between < 0.001 and 0.05), except 5-year-old children and 6-year-old children (mean difference = −0.31, *p* = 1). Finally, behavioral empathy differed between 3-year-old children and the other age groups (mean difference between −1.19 to −1.94, *p* between < 0.001 and 0.002). However, the three other age groups did not differ between each other (mean difference between −0.09 to −0.75, *p* between 0.180 and < 1).

### 5.3. Hierarchical Cluster Analysis of Cases

**Figure 1:**
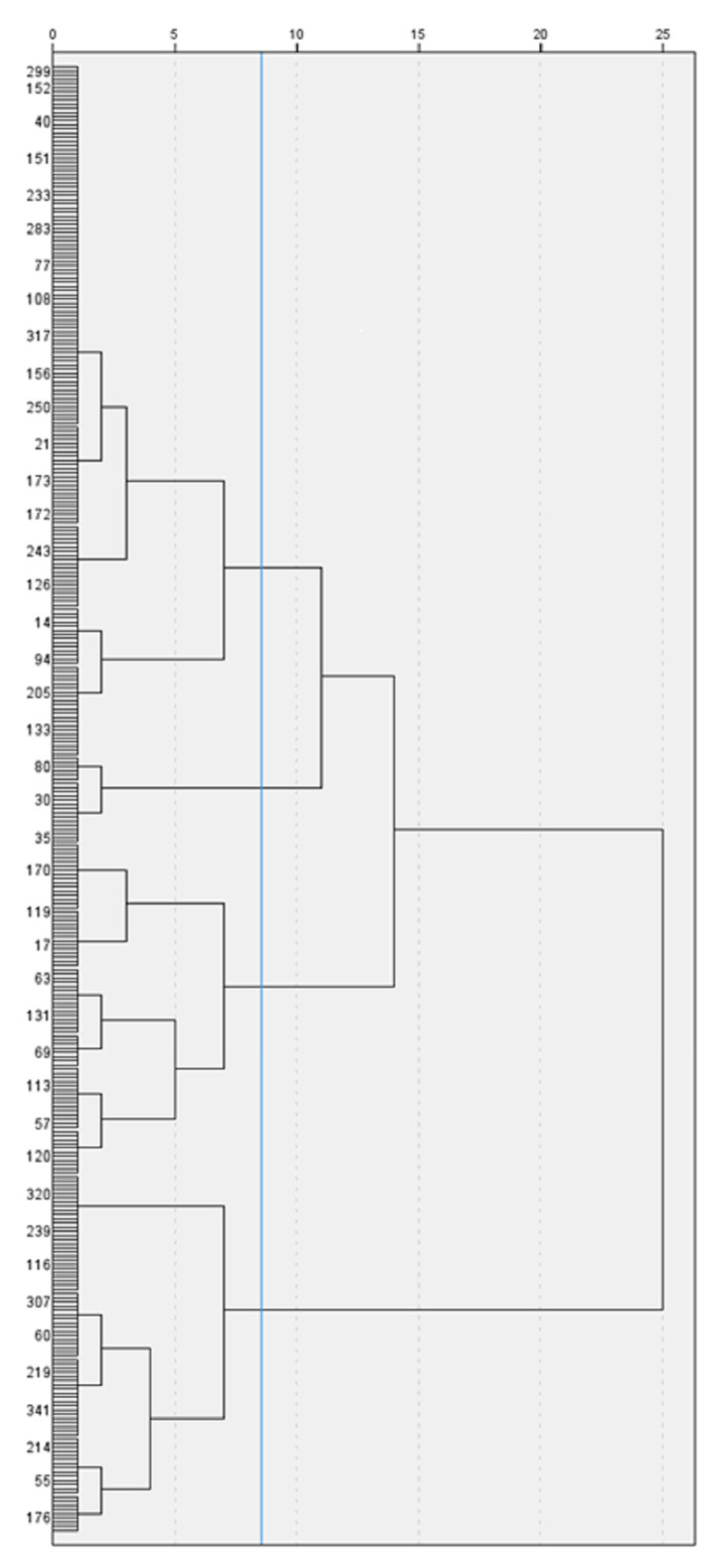
Dendrogram with added line indicating suggested stopping location, resulting from hierarchical cluster analysis, using Ward’s method and Euclidean distance, based on empathy as assessed by hetero-reported questionnaires.

The first hierarchical cluster analysis, comprising the three variables from the questionnaires completed by the mothers, revealed three groups, as represented on the dendrogram (see Figure 1). The average distance between the clusters was 1059.

**Figure 2:**
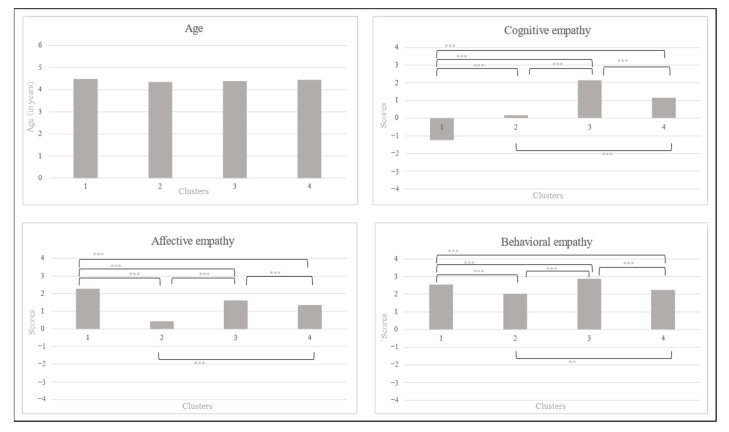
Graphical representations of the descriptive statistics of parental perception of children’s empathy included in each cluster. Notes: ** *p <* 0.01, *** p < 0.001.

Table 4 shows descriptive statistics for the four clusters, as well as the indicators of the one-way MANOVA performed to differentiate the groups. Figure 2 shows graphs illustrating the mean scores of the four clusters of children. The one-way MANOVA indicated a significant effect of group (Pillai’s *F* = 53.826, *p* < 0.001, *η_p_*^2^ = 0.382). More specifically, a significant effect of group appeared concerning affective empathy (*F* = 74.688, *p* < 0.001, *η_p_*^2^ = 0.390). Bonferroni’s post-hoc analysis showed that all groups differed from each other in affective empathy (*p* between < 0.001 and 0.007). Regarding cognitive empathy, a significant effect of group also appeared (*F* = 122.136, *p* < 0.001, *η_p_*^2^ = 0.511), and Bonferroni’s post-hoc test revealed differences between all groups (*p* = < 0.001). Finally, a significant effect of groups also emerged for behavioral empathy (*F* = 78.875, *p* < 0.001, *η_p_*^2^ = 0.403), with significant differences between the four clusters revealed by post-hoc analyses using Bonferroni correction (*p* between < 0.001 and 0.004). Age was also included in the analysis, but showed no difference between groups (*F = 0*.175, *p* = 0.913, *η_p_*^2^ = 0.001). More concretely, Cluster 3 seemed to be composed of children with high scores in the three dimensions of empathy, while Cluster 2 included children with weaker skills in regards to affective, cognitive, and behavioral empathy, in comparison with the other groups. Children included in Cluster 1 seemed to exhibit high levels of affective and behavioral empathy, but some difficulties in cognitive empathy. Finally, Cluster 4 seemed to be an intermediate group of children, who showed moderate levels of skills in the three dimensions of empathy.

**Figure 3:**
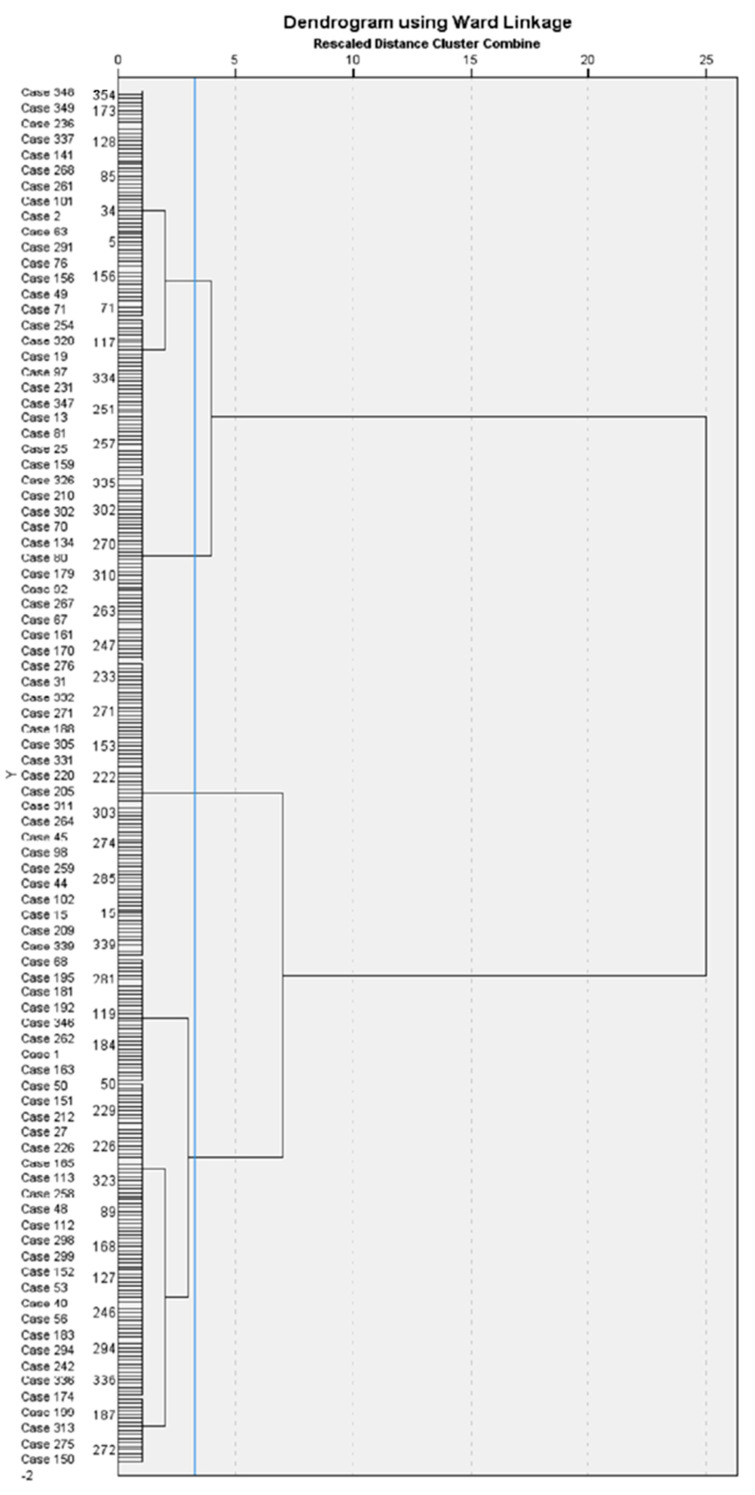
Dendrogram, with added line indicating suggested stopping location, resulting from hierarchical cluster analysis, using Ward’s method and Euclidean distance, based on empathy assessed by a performance-based measure.

The second hierarchical cluster analysis, including the three subscales of the empathy task, revealed four groups to explain the differences between children, depending on their empathic skills (see Figure 3 for the dendrogram). The average distance between clusters was 1059.

**Figure 4:**
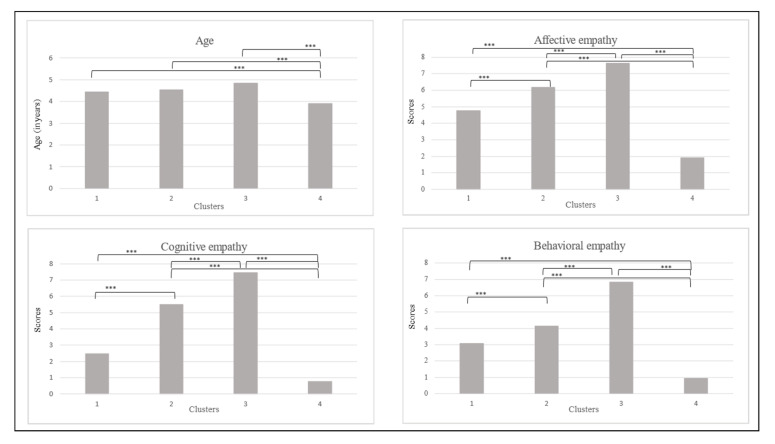
Graphical representations of descriptive statistics for empathy, assessed by a performance-based task, for the children included in each cluster. Notes: *** *p* < 0.001.

Table 5 shows the descriptive statistics and the indicators that emerged from the one-way MANOVA. These four clusters are visually represented in Figure 4. A significant effect of group was demonstrated in the analysis (Pillai’s *F* = 53.729, *p* < 0.001, *η_p_*^2^ = 0.381). More precisely, a significant effect of group appeared in each subscale of empathy (*F* = 368.294, *p* < 0.001, *η_p_*^2^ = 0.759 for affective empathy, *F* = 502.029, *p* < 0.001, *η_p_*^2^ = 0.811 for cognitive empathy, *F* = 150.564, *p* < 0.001, *η_p_*^2^ = 0.563 for behavioral empathy). Bonferroni’s post-hoc analyses highlighted differences between all clusters for the three empathy dimensions (*p <* 0.001). Age was also included in the one-way MANOVA and showed a significant effect of group (*F* = 10.255, *p* < 0.001, *η_p_*^2^ = 0.081). However, Bonferroni’s post-hoc test revealed that only Cluster 4 differed from the three other groups, according to age (*p* < 0.001). Clusters 1, 2, and 3 did not differ between each other (*p* between 0.080 and 1). In terms of the groups’ empathy profiles, Cluster 3 included children with higher scores in the empathy task, while Cluster 4 integrated children with lower empathy scores. However, the children in Cluster 3 were the older children, and those in Cluster 4 were the younger preschoolers. The children in Cluster 1 were those with average scores for affective empathy, but with below-average scores for cognitive and behavioral empathy. Finally, Cluster 2 comprised children with an average level of empathy in the three subscales.

## 6. Study 2

### 6.1. Objective

This longitudinal study explored the developmental trajectory and predictive links between affective, cognitive, and behavioral empathy in preschoolers over one year. It was hypothesized that the three subscales at Time 2 (one year later) would be predicted by themselves and by the two other empathic dimensions at Time 1. Thus, the strengths or weaknesses in each dimension of empathy at Time 1 might or might not be stable at Time 2. Moreover, concerning the intensity of the links between the three dimensions of empathy at each time of measurement, it was expected that the inter-relational dynamic might differ (through covariances at Time 1 and at Time 2).

### 6.2. Participants

On the basis of the same inclusion criteria as those used in the first study, 69 preschoolers were evaluated twice within one year (age at Time 1: *M =* 3.90, SD = 0.77; age at Time 2: *M =* 4.94, SD *=* 0.80). The same demographic information as that used for Study 1 was collected to identify the characteristics of the sample. Concerning family situation, 89.1% of the children lived with both parents, while the remaining 10.1% lived alternately with their mother and their father. A total of 13.8% of the children were only children, 52.3% had one sibling, and the remaining 33.8% had two or more siblings. The mothers had an average level of education equivalent to a bachelor’s degree, and the fathers were educated, on average, to a high school level. The average family income was EUR 4000 to 4500 per month.

### 6.3. Measures

The measures were the same measures used in Study 1.

### 6.4. Procedure

The procedure for recruiting children was identical to that of the first study. At Time 1, the empathy task was administered to children, and the mothers completed the GEM-vf and the prosocial actions scale of the EmQue-vf. One year later, at Time 2, the same performance-based measure was administered, and the same hetero-reported questionnaires were completed. After each evaluation, the children and mothers received a small gift to thank them for their participation in this study.

## 7. Data Analysis

As in Study 1, a missing data analysis was first performed to improve the statistical power, using IBM SPSS 29 (IBM Corporation, Armonk, NY, USA). Subsequently, two cross-lagged models were estimated, using maximum likelihood (ML) in Stata 17, to estimate the relationship between the affective, cognitive, and behavioral empathic skills at Time 1 and those at Time 2. The covariances between the three empathic dimensions at each time of measurement were also considered in order to explore the inter-relational dynamic of the links. To assess the goodness-of-fit, the root mean square error of approximation (RMSEA) was required to be below 0.05, the comparative fit index (CFI) and Tucker-Lewis index (TLI) had to be above 0.95, and the standardized root mean square residual (SRMR) had to be below 0.08, as recommended by [32] Additionally, each model’s R-squared (*R*^2^) values were reported, along with the identification of significant paths between the dependent and independent variables.

## 8. Results 

### 8.1. Preliminary Analysis

The empathy variables of the questionnaires and the performance-based analysis were screened to improve the statistical power through a missing data analysis. The percentage of missing data across the variables at the two time points was 22.3%, on average (range = 8.7–40.6%). Moreover, the pattern of missingness was completely random (Little’s MCAR test: χ^2^ = 232.41, DF = 246, *p* = 0.728). Missing values were therefore imputed by using expectation maximization imputation. Based on the skewness and kurtosis values, analyses of the variables then showed that the data followed a normal distribution. Furthermore, using the variance inflation index (VIF), no multicollinearity was observed. Finally, Table 6 presents the descriptive statistics of this longitudinal study.

### 8.2. Cross-Lagged Analysis

**Figure 5:**
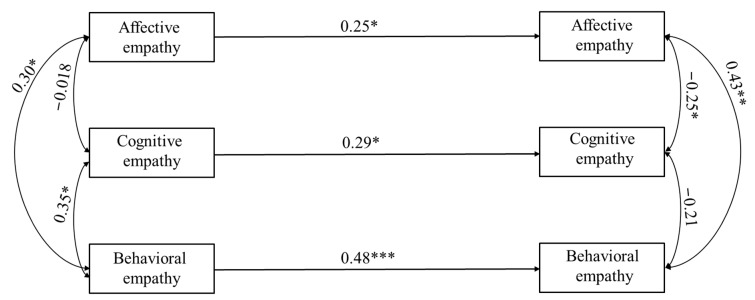
Paths from children’s empathic skills at Time 1 to children’s empathic skills at Time 2, assessed by the hetero-reported questionnaire. Notes: * p < 0.05; ** *p* < 0.01; *** *p* < 0.001.

The first of the two models was constructed using the hetero-reported questionnaires completed by the mothers, while the second concerned the performance-based measure. Figure 5 shows how empathic skills, as perceived by the mothers, at Time 1 were predictively related to the same variables one year later (at Time 2). Only the significant paths are represented in the figure. This model was saturated (RMSEA = 0.000, CFI = 1, TLI = 1, SRMR = 0.000). The *R*^2^ of the complete model was 0.33. According to the results, affective empathy at Time 2 was explained by 8% of the variance in predictive variables and was only predicted by affective empathy at Time 1 (coefficient = 0.18, z = 2.07, *p* = 0.038). Concerning cognitive empathy, the path coefficient value between this variable at Time 1 and Time 2 was 0.25 (z = 2.41, *p* = 0.016, *R*^2^ = 0.10). The *R*^2^ related to behavioral empathy at Time 2 was 0.19. Moreover, the path coefficient for the relationship between behavioral empathy at Time 1 and Time 2 was 0.31 (z = 3.94, *p* < 0.001). Figure 3 also shows the covariance between variables at each time point. At Time 1, both affective and cognitive empathy positively covaried with behavioral empathy (coefficient between 0.13 and 0.17, *z* between 2.38 and 2.72, *p* between 0.007 and 0.018). At Time 2, the covariance between affective empathy and cognitive empathy was negatively significant (coefficient = −0.12, *z* = −1.99, *p* = 0.047), while that between affective empathy and behavioral empathy was positively significant (coefficient = 0.078, *z* = 3.28, *p* = 0.001). Table 7 and Table 8 show the coefficients and covariances of the whole model. The second model regarding the performance-based measure showed no significant results.

## 9. Discussion

Through two studies, an attempt was made to improve the understanding of preschoolers’ empathic skills and their developmental trajectory within one year, using two methods of assessment. In this way, several objectives were pursued to examine the role of gender and age in the development of empathy, as well as whether any distinct profiles of skills in regards to affective, cognitive, and behavioral empathy stood out in a subgroup of children aged 3 to 6 years, depending on their skills in the three dimensions of empathy. The longitudinal study was intended to assess how empathic skills at a given time predicted these same empathic skills one year later.

First, in the analysis, no difference in children’s empathic skills, as perceived by the mothers, appeared to be gender-dependent, regardless of the dimension. This corroborated the findings of past studies showing that boys and girls did not differ in their level of affective [7,8,13], cognitive [7,8,20], or behavioral [14] empathy. Contrary to the findings of Strauss [12], these results showed that children’s empathic skills expressed in real-life social situations, as reported by their mothers, were not influenced by the role of gender, which could be implicitly learned from early childhood. Currently, parents appear to raise boys and girls in a more gender-neutral way and to be more supportive of their children’s emotions in reaction to the emotions of another person. However, when the three dimensions of empathy were assessed through a performance-based measure, it was found that girls tended to have better affective empathy than boys. In other words, preschool girls were more able than boys to identify and feel the same emotion as the character in the story. Using a task based on the children’s performance, rather than a hetero-reported questionnaire, only Strayer and Roberts [10] obtained a difference between boys and girls in regards to empathy. However, in their study, affective and cognitive empathy were aggregated to produce a global empathy score, making it difficult to establish whether the difference concerned affective or cognitive empathy.

Concerning the relationship between empathic skills and age, the result of the first study highlighted an increase in children’s empathy during the preschool period as they grew older, with both the correlation coefficients and the one-way MANOVA showing that empathy was weaker in younger children than in older children, within the targeted range of 3 to 6 years. This is in line with past studies which demonstrated an increase in empathy with age, in the affective, e.g., [7,20], cognitive, e.g., [13,15], and behavioral [15,24] dimensions. This result applied to the three subscales of the performance-based measure of empathy, meaning that explicit empathy improved. This explicit empathy corresponds to the direct responses of children to a hypothetical emotional situation illustrated in pictures or videos. With the evolution of their language and cognitive abilities, children were increasingly able to feel others’ emotions, to understand why they felt a particular way, and to engage in effective prosocial behaviors to soothe the emotion of another person. Surprisingly, when parental perception was considered, a stagnation in cognitive empathy seemed to appear, while an increase in affective and behavioral empathy was reported. Although the result regarding the affective and behavioral dimensions was in line with those of past studies [7,20,31,33], the mothers’ perception that cognitive empathy had not increased with age is not consistent with the observations in most of the literature, which has found an evolution in this dimension [7,34]. However, Nader-Grosbois and Simon [20] showed that cognitive empathy did not differ between groups of children aged 3 to 5 years and 6 to 8 years, meaning that it remains stable between preschool age and the beginning of school age. This suggests that mothers could have difficulties in seeing the manifestations of cognitive empathy and inferring the internalized process of their children’s understanding in daily-life situations. In contrast, as externalized reactions to an emotional situation, affective and behavioral empathy are probably more easily identified by mothers. Moreover, concerning the positive relationship between age and behavioral empathy, this means that children could engage in more prosocial actions as they grow older and learn social rules in their everyday environment.

Thanks to hierarchical cluster analysis, the 354 preschoolers were distributed in different groups, depending on their skills in the three dimensions of empathy, as assessed by a hetero-reported questionnaire or a performance-based measure. When parental perception was considered, four groups were identified. Cluster 3 included children who were perceived as having the highest profile of empathy, whatever the dimension. These children were able to feel the emotions of others, to understand why they themselves felt these emotions, and where these emotions came from, as well as to engage in effective prosocial behaviors, considering others’ needs. Conversely, Cluster 2 comprised children who were perceived as having the lowest skill levels in the three dimensions of empathy. However, their implicit behavioral empathy showed an average skill level, indicating that these children were able to respond to others’ emotions in an appropriate way, independently of their affective and cognitive empathy. Cluster 4 integrated children perceived as having a more moderate profile of empathy. Their empathic skills were more apparent in some specific situations than in others. Children included in Cluster 1 presented a specific profile of empathy: they were viewed as having a high level of affective empathy, but also as having difficulties regarding cognitive empathy. Their high level of affective empathy may have created an effect of emotional contagion, which prevented them from understanding the emotional situation experienced by the other person. Like the children in Cluster 2, the children of Cluster 1 were perceived as having an average level of behavioral empathy. It is important to note that these differences in empathic skills between these clusters appeared, regardless of age. Thus, although previous studies [7,13,15,20,24] and the present results regarding age suggest that empathic skills develop as children grow older, some children were identified as having difficulties in any dimension, whatever their age. Therefore, these children with empathic difficulties could be helped, thanks to the implementation of intervention (individually or in a group) to prevent them from developing social problems (such as aggressivity, opposition, or bullying behaviors), as was demonstrated in previous studies regarding the link between empathy and social adjustment [11,14].

Based on the subscales of the performance-based measure, the second hierarchical cluster analysis of cases also identified four groups of children according to their explicit empathic skills. Cluster 3 concerned children who performed better in the empathic task. This group of children also largely consisted of the oldest preschoolers. They were able to identify their own emotions in reaction to the emotion of the character of the story, to explain why they felt this emotion, and to give some examples of prosocial behaviors they could engage in to soothe the emotion of the character. Conversely, Cluster 4 consisted of children with the weakest performance on the three subscales of empathy; they were also the youngest children. These two groups confirm that children’s empathic skills improve with age, as reported previously [7,13,15,20,24]. As success in this performance-based measure depended on cognitive and language development [29,35], the children in Cluster 4, whose average age was less than 4 years, may not yet have had sufficient abilities to answer the questions. The children in Cluster 2 achieved an average level on all the three subscales of empathy, but their mean age was the same as that of Cluster 3. The last group, Cluster 1, contained children with an average score for affective empathy, but with some difficulties in regard to cognitive and behavioral empathy. As affective empathy consists of an automatic reaction [3], it may be easier for some children to identify what they are feeling than to explain why their emotion has arisen and what prosocial behaviors they can engage in, which involves the use of two controlled processes [3].

The second study aimed to assess how children’s empathic skills at a given time could predict the same skills one year later. As expected, each empathic dimension, as perceived by the mothers, positively predicted the same dimension one year later. However, no relationship between the different dimensions at Time 1 and Time 2 was found. Therefore, at preschool age, neither affective, cognitive, nor behavioral empathy predicted the two other dimensions one year later. Covariance between the three dimensions at each time point showed a positive relationship between affective and behavioral empathy (at Time 1 and Time 2), as well as between cognitive and behavioral empathy (at Time 1 only), as was also the case in the research of Nader-Grosbois and Simon [20]. In other words, being emotionally affected by, or understanding the emotion of others could help children to engage in behaviors to soothe them. However, contrary to the study of Nader-Grosbois and Simon [20], affective and cognitive empathy were not related at Time 1 and were negatively related at Time 2. According to Heyes [3], cognitive empathy could be activated without the person feeling the other’s emotion. Moreover, as affective empathy is an automatic process [3], emotional reactions to another’s distress could be activated without the child understanding the emotional situation experienced by the other person. Furthermore, excessive emotional contagion could prevent children from understanding the emotional situation experienced by that person. However, the relationship between affective and cognitive empathy remains difficult to understand, given the results of the studies conducted to date, which were not always convergent with the theoretical models of empathy. As suggested by Bulgarelli and Jones [30], new models of empathy, questioning the relationship between affective and cognitive empathy, should be tested on children in a more ecological setting.

Although the results of these two studies add nuances to the literature on empathy at preschool age in terms of subgroups identified according to empathy level in the three dimensions and on the developmental trajectory of empathy at preschool age, some limitations can be identified. The first concerns the high level of socioeconomic status of the participants. A high proportion of parents had a high level of education, equivalent to a bachelor’s or master’s degree. It could be relevant to replicate these two studies with more diversity in the sample. The second limitation concerns the one-year interval of time in the longitudinal study, which could be too short to observe a developmental change in empathic skills, depending on the three dimensions. The interval of time could be extended to two years in future studies in order to examine the possible development of the children’s profiles. The third limitation concerns the internal consistency of cognitive empathy, as assessed by the GEM-vf. Its limited Cronbach’s alpha, also found in the French validation study, could be explained by the fact that the subscale is composed of only four items. Consequently, the results relating to cognitive empathy should be interpreted with caution. The fourth limitation concerns the evaluation of affective empathy. Physical and physiological reactions are an important component of affective empathy, but the measures used in these two studies were only based on the parents’ perception of their children’s reactions, or the identification of emotions by the children themselves. Including an objective measure of the children’s physiological reactions (such as pupillometry, heart rate, or skin conductance) in response to others’ emotions could yield relevant information. In addition, questionnaires regarding empathy were only filled in by mothers, which may have biased the results. The replication of this type of study with multiple informants could produce more robust statistics. The final limitation concerns the missing data, which may have impacted the analyses. Although parents made a commitment to help with the research, the proportion of uncompleted questionnaires was 17% and 22%, respectively, in the two studies. Mothers may have had difficulty understanding the questions or applying them to their children. They may also have been uncomfortable in certain situations in which they imagined their children’s reaction to be socially undesirable. However, as the percentage of missing data was above 5%, but the pattern of missingness was completely random, data imputation was performed to improve the power of the statistical analyses, as suggested by Madley-Dowd, Hughes [36].

These studies raise new questions about preschoolers’ empathic development. Future studies should investigate the link between the three dimensions of empathy and other developmental domains, such as the theory of mind, emotional regulation, or social adjustment. Regarding the distinct profile of children at preschool age, it would make sense to investigate the effect of individual or group training programs on children presenting difficulties in regards to empathy. Moreover, carrying out this type of study on populations with atypical development (e.g., those with intellectual disabilities, autism spectrum disorder, or externalized behavior disorder) would seem to be a relevant approach to improving prevention and interventions.

## 10. Conclusions

This paper highlights new contributions to our understanding of the development of empathy. Beyond the effect of gender and age on the three dimensions of empathy and their predictability one year later, the results revealed the diversity of empathic profiles at preschool age. It was found that some children, aged between 3 to 6 years, appeared to have difficulties in one or several dimensions, which could impact their emotional and social development. It would therefore make sense to pay attention to children who may be overwhelmed by other people’s distress or emotions. Helping these children to identify their emotions, as well as to understand emotional situations and to add to their repertoire of prosocial behaviors, could benefit their relationships with other children or adults. Given the differing results, depending on the type of measure used, it would be useful both to administer performance-based measures to children and to include hetero-reported questionnaires completed by both parents or another familiar adult in order to assess their needs in terms of the development of empathic skills.

## Figures and Tables

**Table 1:** Demographic characteristics of participants’ families.

	*M* (SD)	%
Mothers’ education level	5.09 (1.16)	
Primary school		2.4%
Vocational qualification		9.1%
High school qualification		14.0%
Bachelor’s degree		31.1%
Master’s degree		39.5%
Ph.D.		3.9%
Fathers’ education level	4.68 (1.31)	
Primary school		3.2%
Vocational qualification		21.1%
High school qualification		18.2%
Bachelor’s degree		21.1%
Master’s degree		34.6%
Ph.D.		1.8%
Family income	8.01 (2.53)	

**Table 2:** Descriptive statistics.

	Total			Boys			Girls		
	*M* (SD)	Skewness	Kurtosis	*M* (SD)	Skewness	Kurtosis	*M* (SD)	Skewness	Kurtosis
N	354			159			205		
Chronological age (in years)	4.40 (1.02)	0.069	−1.140	4.36 (1.025)	0.177	−1.112	4.43 (1.02)	−0.014	−1.141
Hetero-reported measure									
Affective empathy (max = 4)	1.21 (.88)	−0.028	0.98	1.18 (.97)	−0.434	1.379	1.22 (0.81)	−0.064	0.100
Cognitive empathy (max = 4)	1.00 (1.24)	−0.435	0.527	1.08 (1.29)	−0.334	0.104	0.93 (1.20)	−0.565	0.947
Prosocial actions (max = 16)	2.34 (0.49)	0.076	0.627	2.33 (0.49)	0.065	0.544	2.36 (0.50)	0.081	0.725
Performance-based measure									
Affective empathy (max = 8)	4.93 (2.11)	−0.295	−0.693	4.68 (2.09)	−0.052	−1.007	5.12 (2.11)	−0.492	−0.314
Cognitive empathy (max = 8)	3.63 (2.55)	0.126	−1.133	3.45 (2.52)	0.253	−1.111	3.78 (2.57)	0.029	−1.116
Behavioral empathy (max = 8)	3.43 (2.34)	0.076	0.627	3.26 (2.40)	0.263	−1.096	3.57 (2.31)	0.164	−0.782

**Table 3:** Pearson’s inter-correlations between age and empathy skills in both measures.

	Age	Affective Empathy—GEM-vf	Cognitive Empathy—GEM-vf	Prosocial Actions—EmQue-vf	Affective Empathy—Task	Cognitive Empathy—Task
Affective empathy—GEM-vf	0.162 **					
Cognitive empathy—GEM-vf	0.067	0.172 ***				
Prosocial actions—GEM-vf	0.180 ***	0.330 ***	0.164 *			
Affective empathy—Task	0.221 ***	0.039	0.011	0.095		
Cognitive empathy—Task	0.342 ***	0.043	−0.059	0.060	0.744 ***	
Behavioral empathy—Task	0.299 ***	−0.038	0.047	0.059	0.600 ***	0.668 ***

Notes: * p < 0.05; ** *p* < 0.01; *** *p* < 0.001.

**Table 4:** Descriptive statistics of the clusters obtained through hierarchical cluster analysis according to empathy, as perceived by the mothers.

	Cluster 1	Cluster 2	Cluster 3	Cluster 4		
	*M* (SD)	*M* (SD)	*M* (SD)	*M* (SD)	*F*	*η_p_* ^2^
N	21	80	86	167		
Chronological age	4.48 (1.03)	4.35 (1.057)	4.38 (0.984)	4.44 (1.044)	0.175	0.001
Empathy						
GEM-vf—Affective empathy (max = 4)	2.24 (0.48)	0.42 (0.62)	1.59 (0.84)	1.32 (0.48)	74.688 ***	0.390
GEM-vf—Cognitive empathy (max = 4)	−1.21 (0.91)	0.13 (1.27)	2.12 (0.83)	1.13 (0.58)	122.136 ***	0.511
EmQue-vf—Prosocial actions (max = 16)	2.53 (0.40)	2.02 (0.48)	2.85 (0.38)	2.23 (0.30)	78.875 ***	0.403

Notes: *** *p* < 0.001.

**Table 5:** Descriptive statistics of the clusters, obtained through hierarchical cluster analysis, regarding scores on the Empathy Task test.

	Cluster 1	Cluster 2	Cluster 3	Cluster 4		
	*M* (SD)	*M* (SD)	*M* (SD)	*M* (SD)	*F*	*η_p_* ^2^
N	130	100	48	76		
Chronological age	4.44 (1.11)	4.53 (0.98)	4.85 (0.74)	3.91 (0.89)	10.255 ***	0.081
Empathy						
Empathy Task—Affective empathy (Max = 8)	4.76 (1.27)	6.18 (0.95)	7.64 (0.52)	1.90 (0.96)	368.294 ***	0.759
Empathy Task—Cognitive empathy (Max = 8)	2.48 (1.31)	5.49 (1.12)	7.45 (0.65)	0.77 (0.88)	502.029 ***	0.811
Empathy Task—Behavioral empathy (Max = 8)	3.06 (1.80)	4.13 (1.80)	6.83 (0.87)	0.94 (0.93)	150.564 ***	0.563

Notes:; *** *p* < 0.001.

**Table 6:** Descriptive statistics of the longitudinal study.

	Time 1			Time 2		
	*M* (SD)	Skewness	Kurtosis	*M* (SD)	Skewness	Kurtosis
N	69			69		
Chronological age (in years)	3.90 (0.77)	0.291	−1.301	4.94 (0.80)	0.111	−1.428
Hetero-reported measure						
Affective empathy (max = 4)	0.99 (1.01)	−0.109	−0.242	(0.97)	−0.434	1.379
Cognitive empathy (max = 4)	1.01 (1.20)	−0.551	0.140	1.08 (1.29)	−0.334	0.104
Prosocial actions (max = 16)	2.18 (0.61)	0.257	0.500	2.33 (0.49)	0.065	0.544
Performance-based measure						
Affective empathy (max = 8)	4.63 (2.14)	−0.126	−0.829	5.46 (0.1.93)	−0.271	−0.654
Cognitive empathy (max = 8)	3.78 (2.41)	0.172	−1.046	4.5 (2.34)	−0.162	−0.847
Behavioral empathy (max = 8)	3.58 (2.59)	0.163	−1.308	4.17 (2.53)	0.360	−0.018

**Table 7:** All coefficients of paths of the cross-lagged analysis, including the hetero-reported questionnaires.

	*B*	*SE*	*β*	*z*	*p*	*R* ^2^
Affective empathy T2						0.08
Affective empathy T1	0.18	0.087	0.25	2.07	0.038	
Cognitive empathy T1	−0.055	0.077	−0.089	−0.72	0.472	
Behavioral empathy T1	0.089	0.157	0.073	0.567	0.571	
Cognitive empathy T2						0.10
Affective empathy T1	−0.146	0.12	−0.146	−1.22	0.222	
Cognitive empathy T1	0.256	0.106	0.293	2.41	0.016	
Behavioral empathy T1	−0.19	0.215	−0.113	−0.88	0.376	
Behavioral empathy T2						0.19
Affective empathy T1	−0.021	0.044	−0.055	−0.49	0.627	
Cognitive empathy T1	−0.026	0.039	−0.07	−0.68	0.497	
Behavioral empathy T1	0.315	0.080	0.478	3.94	0.000	

**Table 8:** All coefficients of covariances of the cross-lagged analysis, including the hetero-reported questionnaires.

	*Cov*	*SE*	*p*
Time 1			
Affective empathy and cognitive empathy	−0.015	0.103	0.883
Affective empathy and behavioral empathy	0.132	0.103	0.018
Cognitive empathy and behavioral empathy	0.176	0.065	0.007
Time 2			
Affective empathy and cognitive empathy	−0.12	0.06	0.047
Affective empathy and behavioral empathy	0.078	0.023	0.001
Cognitive empathy and behavioral empathy	−0052	0.030	0.085

## Data Availability

The data presented in this study are available on reasonable request from the corresponding author.
